# Attentional Resources and Independence in Basic and Instrumental Activities of Daily Living in Individuals with Intellectual Disabilities

**DOI:** 10.3390/healthcare12020126

**Published:** 2024-01-05

**Authors:** Beatriz García-Pintor, Francisco Manuel Morales-Rodríguez, José Manuel Pérez-Mármol

**Affiliations:** 1 Association in Favor of People with Intellectual Disability—ASPROGRADES, 18007 Granada, Spain; beatrizpintor@gmail.com; 2 Department of Physiotherapy, Faculty of Health Sciences, University of Granada, 18011 Granada, Spain; 3 Department of Educational and Developmental Psychology, Faculty of Psychology, University of Granada, 18011 Granada, Spain; fmmorales@ugr.es; 4 Instituto de Investigación Biosanitaria ibs., 18012 Granada, Spain

**Keywords:** independence, personal autonomy, activities of daily living, attention, intellectual disability

## Abstract

The relationship between attentional resources and functionality in individuals with intellectual disabilities (IDs) is clinically relevant. This study aimed to examine the possible relationship between the degree of ID and attentional resources, and to evaluate whether attentional resources predict the performance of basic and instrumental activities of daily living (ADL) in individuals with mild and moderate ID. This study, which employed a descriptive, cross-sectional, observational design, was conducted between July 2019 and May 2020. The sample consisted of 166 individuals divided into three groups: moderate ID, mild ID, and those without ID. These groups were compared for attentional functions (*p* < 0.001), obtaining an effect size ranging from medium to large. The results indicated that 40% of the variance in basic ADL performance was explained by the age of the participants, degree of disability, and sustained attention in individuals with ID. Additionally, 64% of the variance in instrumental ADL performance was explained by sustained, divided, and executive attention. Therefore, attentional resources appear to be associated with the performance of basic and instrumental ADL in individuals with mild and moderate ID.

## 1. Introduction

The model of human functioning observes an individual from a multidimensional perspective, including intellectual abilities, adaptive behavior, health, participation, and context [1]. New paradigms of intellectual disability (ID) emphasize the need to provide the necessary support to improve the level of human functioning in these individuals [2]. Based on this, a series of parameters are established, and resources are assigned based on person-centered planning [3]. Human functioning is a construct closely related to performance in activities of daily living (ADL) [4]. Individuals with ID often exhibit limitations in both intellectual functioning and adaptive behavior, which can affect their ADL performance [5]. ADL performance usually allows an individual to develop in different spheres of their life and interact with the external environment (other humans, physical, virtual, and other contexts).

ADL can be classified into two groups, basic activities of daily living (BADL) and instrumental activities of daily living (IADL) [6]. BADL refers to lower-complexity activities related to survival, requiring less cognitive processing and manipulative skills. BADL includes activities, such as eating, showering, and dressing. IADL refers to activities that demanding complex cognitive processing because they require the interrelation of multiple processes, social interaction, and community participation, which usually involve caring for others, the use of new technologies, or the use of public transport [7]. According to the International Classification of Functionality, the correct performance of ADL consists of the coordination of function, body structure, activity, and participation. Therefore, impairment or poor development of cognitive processing may negatively impact ADL performance [6].

Attentional cognitive processing refers to the capability for handling, storing, and retrieving information during an activity [8,9]. According to the functional multidimensional model of attention [10,11], attentional resources involve selecting relevant information while suppressing irrelevant information from inner or external contexts [12]. These attentional resources can be divided into five categories. First, the selective attention is defined as the ability to select relevant information while inhibiting attention to certain distracting stimuli. Second, sustained attention is defined as the ability to maintain responses over time. Third, divided attention is defined as the ability to attend to two stimuli simultaneously. Fourth, alternating attention is defined as the ability to change tasks based on the contextual requirements. Alternating attention is closely related to flexibility and may be present in IADL execution when it is necessary to continuously change the focus of attention between mental or physical tasks. Fifth, executive attention functions as an interrelation between other cognitive processes, such as working memory, behavioral inhibition, and self-regulation [8].

Individuals with ID often exhibit high rates of comorbidity with other developmental disorders such as autism spectrum disorder and attentional deficits. Therefore, it is crucial to differentiate whether alterations in attentional processing stem from attention deficits or attentional resource limitations [13]. In both attention deficits and attentional resource limitations, individuals may find it challenging to maintain the regulatory status necessary to perform daily tasks, compared to those without intellectual disabilities [14]. Attentional resources are interrelated with other cognitive processes, such as memory, inhibition, and planning [3,8]. Previous studies indicated that attentional resources, such as planning, self-correction, decision making, and judgment, are necessary for the correct performance of ADL [15,16]. However, these studies were conducted on patients with brain damage and degenerative diseases. To the best of our knowledge, the relationship between attentional resources and functionality in both BADL and IADL in individuals with intellectual disabilities has not yet been examined. These hypotheses should be considered when developing cognitive training programs that can enhance the participation of individuals with ID in their ADL. Training programs could be more effective when the characteristics of the population, cognitive abilities, and limitations of information processing are thoroughly understood.

This study aimed (1) to explore the relationship between the degree of ID and attentional resources, and (2) to evaluate whether attentional resources predict the performance of basic and instrumental ADL in individuals with mild and moderate ID.

## 2. Material and Methods

### 2.1. Design

This study used a descriptive, cross-sectional, observational design. The local Ethics Committee of CEI-Granada (Granada, Spain) approved the study protocol.

### 2.2. Participants

**Figure 1:**
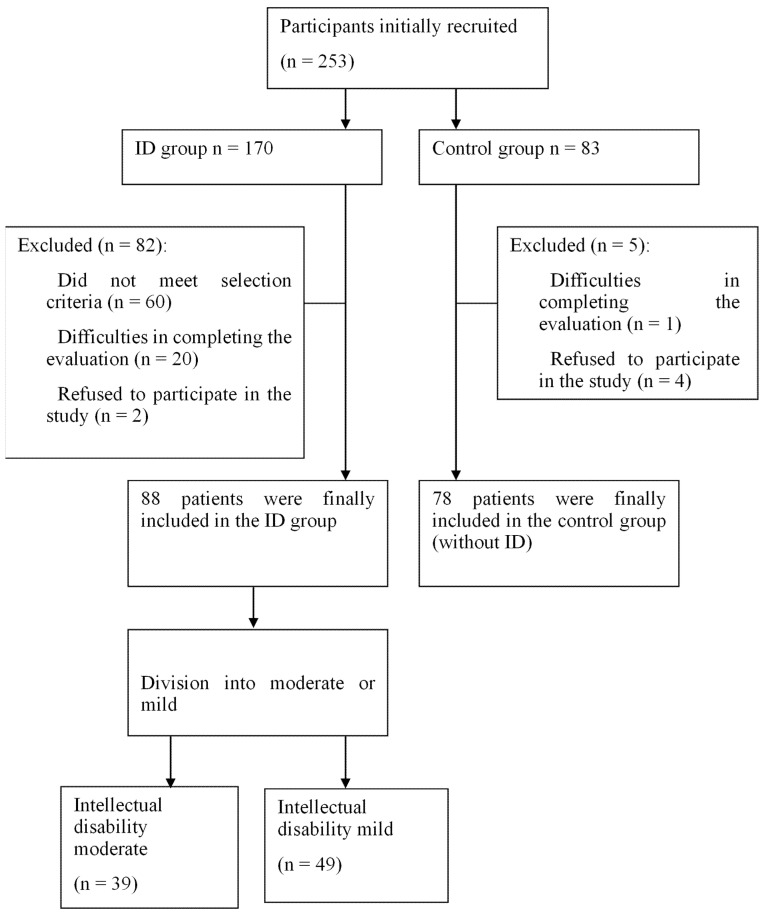
Flowchart illustrating participant selection for this study.

The initial sample consisted of 253 individuals from Granada province, Spain. The analyzed sample, after applying the selection criteria, consisted of 166 participants. The study sample comprised three groups. The group with moderate ID consisted of 39 adults (20 women and 19 men), with an average age of 41.21 years; the group with mild ID comprised 49 adults (21 women and 28 men), with an average age of 39.31 years. The group without ID comprised 78 adults (42 women and 36 men), with an average age of 37.01 years. Participants were recruited from three ID associations and community services (community centers). Figure 1 illustrates the sample selection procedure.

Regarding the selection criteria, the general inclusion criteria for the three groups were (1) >18 years of age, and (2) voluntary participation. The additional criteria for the ID group were (1) having a diagnosis of ID that met the criteria of the World Health Organization [17], and (2) having a moderate or mild degree of ID according to the evaluation of the assessment and guidance center of the Autonomous Government of Andalusia [18].

The exclusion criteria for both groups were as follows: (1) presenting behavioral disturbances, (2) suffering from a diagnosed mental disorder, (3) presenting severe language impairments, in both comprehension and expression, (4) presenting cognitive impairment (through a score of ≥24 points in the Mini-Examination Cognitive of Lobo’s test), (5) mother tongue other than Spanish, (6) being >65 years of age, (7) diagnosed with a neurodegenerative disorder, and (8) uncorrected sensory impairments (vision or hearing).

### 2.3. Data Collection

The evaluation sessions included individual assessments for each participant, which comprised two parts. First, participants were informed concerning the study and its purpose, with the assurance that they could discontinue their participation at any time. The second part involved the administration of the evaluation instruments and lasted for approximately 20–50 min, with an average duration of 30 min. To assess attentional resources, the evaluation room was well lit and free from noise. The information necessary for the study was collected by reviewing the records from the center. Several instruments used in this study were language-free, making them more suitable for cross-cultural and special-needs contexts. The researchers were trained to ensure the consistency and integrity of data collection. The degree of ID was obtained from each participant’s medical records.

Sociodemographic data were obtained regarding age, sex, completed studies, years of education, and literacy. In addition, clinical data, such as manual dexterity and sensory status (including touch sensation, vision, and hearing), were recorded.

The 10-item Barthel index was used for BADL, and the total score ranged from 0 to 100 points, with a higher score indicating greater independence. This scale was validated for the Spanish population, obtaining a Cronbach’s alpha of 0.86–0.92 [19,20]. The Lawton and Brody scale, which is composed of eight items, was used to assess independence in IADL. The score ranges from 0 to 8, with the former being the level of maximum dependence and the latter being the level of total independence [21,22]. This scale was validated for the Spanish population, confirming its reliability and validity, with a Cronbach’s alpha coefficient of 0.70 [23]. Both scales were implemented in two concurrent formats: (i) an informant-based survey administered by the occupational therapist at the institution, who met the criterion of being acquainted with the participant for a minimum of three months before the assessment, assessing the individual’s capability to engage in these activities, and (ii) direct observation of the participants’ functional level during the execution of these activities.

The evaluation of attentional resources was structured according to a functional multidimensional model of attention [10]. The instruments used were implemented in a language-neutral version, adapted to ensure proper understanding of the instructions. This adaptation enhances its applicability in cross-cultural and special-needs contexts. All instruments were administered in a hetero-guided manner in which the evaluator guided the assessment and recorded participants’ performance.

The span of verbal attention was assessed using the “Digits Span” subtest, derived from the Wechsler Adult Intelligence Scale-IV (WAIS-IV) [24]. The maximum possible score was 12 points [25]. This subtest exhibited adequate internal consistency in the Spanish population with a Cronbach’s alpha coefficient of 0.73 [26].

The “A” test was used to evaluate sustained attention. This instrument consisted of a series of letters, including the letter A. The participants were requested to tap on the table when they heard the letter A. This test measured the number of correct items and recorded the number of perseverations, omissions, and commissions. More than two errors in this test are usually interpreted as the presence of alterations in sustained attention [27].

The color trial test was used to evaluate divided and alternating attention. This test consisted of two parts: Part A and Part B. A standard score was obtained by correcting the raw scores for age and schooling time, with higher scores indicating greater attention [28,29].

Forward digit, backward digit, and letter-number sequencing were used to assess executive attention. These subtests were conducted using the Wechsler Adult Intelligence Assessment Scale-IV. The maximum possible score for each subtest was 12 points. These subtests show adequate reliability, with a Cronbach’s alpha coefficient of 0.73, and the letter-number sequencing subtest has a Cronbach’s alpha coefficient of 0.62 [26].

### 2.4. Data Analysis

Statistical analyses were performed using the SPSS software (Version 21.0). The Kolmogorov–Smirnov test was used to determine whether the variables showed a normal distribution (*p* > 0.05). A comparison of the three groups for sociodemographic variables was conducted using a one-way analysis of variance (ANOVA) for quantitative variables and a chi-square test for categorical variables. An ANOVA was also employed to assess the potential differences in attentional resources among the groups (moderate, mild, and without ID). Subsequently, a post hoc analysis was performed using the Bonferroni adjustment. The effect size (Cohen’s d) was calculated to assess the magnitude of the differences between the groups. The control group was included in the design to estimate the magnitude of the hypothesized differences between individuals with ID and those without ID as well as between the two degrees of ID. Finally, multiple linear regression analysis was conducted to examine the relationship between attentional resources and performance in both BADL and IADL for individuals with ID.

## 3. Results

The final sample consisted of 166 individuals, of whom 88 had ID and 78 had no ID. The sociodemographic and clinical characteristics of the three groups are presented in Table 1. No differences were observed between the groups concerning any of these variables (*p* > 0.05).

### 3.1. Group Differences in Attentional Resources

The one-way ANOVA analyses indicated differences between the groups (moderate, mild, and no ID) for all attentional resources: digit span (F = 82.87; *p* < 0.001), forward digit test (F = 85.17; *p* < 0.001), correct A test (F = 68.86; *p* < 0.001), test A errors (F = 29.65; *p* < 0.001), test A omission (F = 12.28; *p* < 0.001), commission test (F = 8.87; *p* < 0.001), color trail test part A (F = 48.50; *p* < 0.001), color trail test part B (F = 48.52; *p* < 0.001), backward digit test (F = 46.64; *p* < 0.001), sequencing digits test (F = 65.43; *p* < 0.001), and letter-number sequencing test (F = 66.09; *p* < 0.001). The results of the ANOVA model and mean (SD) scores for attentional resources are presented in Table 2.

Regarding the post hoc comparisons between groups, the results indicated significant differences between individuals with moderate and mild ID for the following attentional variables: digit span (*t* = 7.54, *p* < 0.001), forward digit test (*t* = 2.08, *p* = 0.005), test A errors (*t* = 16.63, *p* < 0.001), test A omission (*t* = 8.27, *p* = 0.001), commission test (*t* = 4.91, *p* = 0.002), backward digit test (*t* = 2.57, *p* < 0.001), sequencing digits test (*t* = 2.49, *p* < 0.001), and letter-number sequencing (*t* = 4.91, *p* = 0.001). These differences ranged in effect sizes (Cohen’s d) from small to large (d = 0.256, 1.154).

The post hoc comparison between the groups of individuals with moderate ID and the group without ID also showed significant differences in all attentional resources: digit span (*t* = 22.33, *p* < 0.001), forward digit test (*t* = 8.01, *p* < 0.001), correct A test (*t* = 26.36, *p* < 0.001), test A errors (*t* = 22.45, *p* < 0.001), test A omission (*t* = 11.64, *p* < 0.001), Test A commission (*t* = 5.87, *p* = 0.001), color trail test part A (*t* = 33.77, *p* < 0.001), color trail test part B (*t* = 35.00, *p* < 0.001) backward digit test (*t* = 6.13, *p* < 0.001), sequencing digits test (*t* = 6.44, *p* < 0.001), and letter-number sequencing (*t* = 14.51, *p* < 0.001). The effect size (Cohen’s d) varied from moderate to large according to the variables (d = 0.761, 4.337).

The post hoc comparison between the mild ID group and the group without ID indicated significant differences in all attentional resources: digit span (*t* = 14.78, *p* < 0.001), forward digit test (*t* = 5.92, *p* < 0.001), correct A test (*t* = 24.51, *p* < 0.001), color trail test part A (*t* = 35.38, *p* < 0.001), color trail test part B (*t* = 34.12, *p* < 0.001), backward digit test (*t* = 3.57, *p* < 0.001), sequencing digits test (*t* = 3.95, *p* < 0.001), and letter-number sequencing (*t* = 9.59, *p* < 0.001). The effect size (Cohen’s d) varied from moderate to large according to the variables (d = 0.616, 2.679). The means (SD) of the results of these measurement variables for the three groups and the effect sizes of the differences between the groups are presented in Table 2 and Table 3.

### 3.2. Association between Attentional Resources and BADL and IADL in Individuals with ID

The linear regression analysis, conducted for the moderate and mild ID groups, indicated that age (in years), degree of disability, and errors on “A test” were predictors of the level of performance on BADL (total scores on the Barthel index). This model explained 40% of the total variance (R^2^ = 0.397) in ID individuals’ ability to perform these activities. Table 4 presents the linear regression analysis results.

The linear regression analysis, conducted on individuals with ID, found that the A test (correct items and mistakes), direct forward, backward digit, and sequencing digits were predictors of the level of performance in IADL (Lawton and Brody scale scores). This model accounted for 68% of the total variance (R^2^ = 0.684) in individuals with ID’s ability to perform these activities. Table 5 presents the linear regression analysis results.

## 4. Discussion

This study aimed to examine the relationship between the degree of ID and attentional resources and to examine whether attentional resources are linked to the performance of BADL and IADL in individuals with mild to moderate ID.

The comparison between individuals with mild and moderate ID revealed differences in the capacity for selective, sustained, and executive attention on a scale ranging from small to large; however, there were no differences among divided and alternating attention. The ID group exhibited an impaired ability to focus on relevant information in contexts with a high number of stimuli. This may indicate a slower speed of attention processing and a decrease in inhibitory control when responding to stimuli. The ability to select relevant information within a given context diminished when there was a high stimulus level. When comparing the groups with ID to those without it, the results showed moderate to large differences in all attentional resources evaluated in this study. These differences may be attributed to individuals with mild ID tending to have fewer opportunities to engage in more complex activities [30]. Performing more complex activities results in improved learning and performance of daily activities [31]. Since these activities are executed daily, constant training positively impacts the development of performance because it promotes cognitive capabilities [6,31]. Similar studies conducted on adolescents and young individuals with ID support this study’s results by showing that adolescents and young individuals with ID exhibit a generalized deficit in executive functions closely related to attentional resources [8,9,32]. This may be explained by an overprotective environment created by their families or institutional care, which limits the development of cognitive processing and the execution of daily living activities [4,33]. A study conducted on children with ID revealed a lower performance in attentional processing during a walking task than in individuals without ID. Given that the walking task involves high attentional demands, this study suggested that these individuals exhibited lower ability to simultaneously perform a secondary task with the same efficiency [34].

Attentional resources appear to be related to the level of ADL performance; that is, the limitation in attentional processing of individuals with ID is associated with dysfunction in these activities, specifically, the age of the participants, degree of disability, and sustained attention explained the BADL performance in the sample with mild to moderate ID. However, the directionality of this association could not be confirmed because the data in the present study were collected cross-sectionally. Older age, higher degree of disability, and lower levels of sustained attention appeared to be related to the level of functioning in BADL. Conversely, the level of functioning could also influence cognitive function. A possible explanation for these results is that these attentional functions are integral to the processes that govern and monitor human behavior, facilitating the interaction of individuals with their environment. In contrast, sustained, divided, and executive attention explained the level of IADL performance in the individuals with ID. This finding may be understood because, based on the definition of IADL, these are tasks that involve greater cognitive processing [6,30]. Therefore, the execution of these activities requires selecting relevant information in the presence of distractors, maintaining attention for a period, the ability to simultaneously focus on two stimuli, or shifting attention to meet the needs of other contexts [6]. Previous studies have reported results similar to those found in this study but with different population samples. A study conducted among older adults living in a community reported an association between attentional demands and the ability to manage ADL. The authors observed that attentional demands were linked to the management of ADL, which required selective attention, such as climbing stairs, furnishing houses, or obtaining information on health procedures [35]. Other investigations, including research on individuals with Alzheimer’s disease [36] and traumatic brain injuries affecting the frontal lobe, also found a relationship between attentional resources and functionality [15]. A study conducted on healthy young individuals revealed how executive functions are related to ADL performance. Executive functions include changing mental attitudes, updating and monitoring information, and inhibiting responses. The results revealed that these functions contribute differentially to the performance of complex tasks [37]. Another study, which is less related to the present study, reported that individuals affected by breast cancer exhibited attentional fatigue as a predictor of their work capacity. Therefore, this population may perform worse on tasks requiring sustained attention at work [38].

### Limitations

Given that the data were collected cross-sectionally, the directionality of the association could not be affirmed. Therefore, although this study has shown different associations between attentional resources and functionality for ADL, the results should be interpreted with caution because of the possible differences regarding other populations and the characteristics of individuals with ID.

## 5. Conclusions

This study revealed differences in the processing of attentional resources among individuals with intellectual disabilities. Distinct variations were observed, not only between the group with ID and those without ID in all attentional resources but also when examining the relationships between individuals with moderate and mild ID. Differences in selective, sustained, and executive attention were identified, whereas no significant differences were observed in divided and alternating attention. Furthermore, attentional resources appeared to significantly impact the performance of both BADL and IADL. Age, degree of disability, and sustained attention predicted 40% of the BADL performance, whereas sustained, divided, and executive attentional resources predicted 64% of the variance in IADL performance.

Individuals with ID exhibit alterations in attentional processing that affect ADL execution, environmental functionality, and overall quality of life. It should be noted that this study was conducted within a context with similar characteristics determined by the geographical area of the collected sample. Nevertheless, the findings have significant implications for the design of intervention programs. Such programs could play a vital role in the development of effective support strategies and training plans tailored to the unique needs of individuals with ID. Ultimately, these interventions have the potential to enhance ADL efficiency and promote greater participation among individuals with ID [3].

## Figures and Tables

**Table 1:** Mean (SD) and frequency (%) for sociodemographic and clinical characteristics of participants with and without ID.

Sociodemographic and Clinical Characteristics	Group with ID *N* = 88		Group without ID *N* = 78	F/Chi-Square (χ^2^)	*p*-Value
	Group with Moderate ID *N* = 39	Group with Mild ID *N* = 49	Mean (SD)/Frequency (%)		
	Mean (SD)/Frequency (%)	Mean (SD)/Frequency (%)			
Age (years)	41.21 (12.15)	39.31 (9.53)	37.01 (14.67)	1.49	0.23
Sex				1.49	0.47
Women	20 (51.3)	21 (42.9)	42 (53.8)		
Men	19 (48.7)	28 (57.1)	36 (46.2)		
Level of education				12.43	0.05
No schooling	2 (5.1)	2 (34.1)	0		
<5 years	6 (15.4)	3 (6.1)	4 (5.1)		
5–10 years	14 (35.9)	17 (34.7)	19 (24.4)		
>10 years	17 (43.6)	27 (55.1)	55 (70.5)		
Handedness				4.04	0.40
Right-handed	33 (84.6)	42 (85.7)	67 (85.9)		
Left-handed	6 (15.4)	5 (10.2)	6 (7.7)		
Ambidextrous	0	2 (4.1)	5 (6.4)		
Touch sensation impairment				3.28	0.19
Yes	1 (2.6)	0	0		
No	38 (97.4)	49 (100)	78 (100)		
Visual impairment				-	-
Yes	0 (0)	0 (0)	0 (0)		
No	39 (100)	49 (100)	78 (100)		
Auditory impairment				-	-
Yes	0 (0)	0 (0)	0 (0)		
No	39 (100)	49 (100)	78 (100)		

**Table 2:** Mean (SD) and group differences in attentional resources among individuals with moderate ID, mild ID, and without ID.

Variable	Group with Moderate ID N = 39	Group with Mild ID N = 49	Group without ID N = 78	F	Lower Limit	Upper Limit	*p*-Value
	Mean (SD)	Mean (SD)	Mean (SD)				
**Span of verbal attention**							
Digit span (total)	5.50 (6.16)	13.04 (7.18)	27.83 (7.37)	82.87 **	12.81	17.16	<0.001
Digit forward test	2.39 (2.78)	4.48 (2.85)	10.40 (2.80)	85.17 **	5.18	6.75	<0.001
**Test A**							
Correct	6.54 (11.03)	8.40 (11.64)	32.90 (6.10)	68.86 **	11.86	17.63	<0.001
Mistakes	22.45 (19.28)	5.82 (9.04)	0.00 (0.00)	29.65 **	6.17	11.90	<0.001
Omission	11.63 (15.17)	3.36 (7.71)	0.00 (0.00)	12.28 **	2.91	6.97	<0.001
Commission	5.87 (10.91)	0.95 (1.49)	0.00 (0.00)	8.87 **	0.89	3.35	<0.001
**Color Trail Test**							
Part A (Standard Score)	56.61 (5.19)	55.00 (0.00)	90.38 (18.67)	48.50 **	70.78	81.30	<0.001
Part B (Standard Score)	56.94 (5.66)	57.83 (6.83)	91.95(18.40)	48.52 **	72.22	82.80	<0.001
Digit backwards	1.81 (2.35)	4.38 (2.53)	7.95 (3.21)	46.64 **	4.29	5.64	<0.001
Digit sequencing	1.21 (1.47)	3.71 (2.91)	7.67(2.55)	65.43 **	3.80	5.11	<0.001
Letters-number sequencing	2.51 (3.84)	7.43 (6.60)	17.02 (5.39)	66.09 **	7.83	10.75	<0.001

Note: ** *p* < 0.001. SD: standard deviation. F: F-value, coefficient of variance.

**Table 3:** Mean (SD) and differences between the moderate ID group and mild ID group, the moderate ID group and the group without ID, and the mild ID group and the group without ID in attentional resources.

Variable	Moderate ID–Mild ID			Moderate ID–Without ID			Mild ID–Without ID		
	*t*	*p*-Value	Cohen d	*t*	*p*-Value	Cohen d	*t*	*p*-Value	Cohen d
**Span of verbal attention**									
Digit span (total)	7.54 **	<0.001	1.127	22.33 **	<0.001	3.288	14.78 **	<0.001	1.127
Digit forward	2.08 *	0.005	0.742	8.01 **	<0.001	2.871	5.92 **	<0.001	2.095
**Test A**									
Correct	1.84	1.00	0.164	26.36 **	<0.001	2.957	24.51 **	<0.001	2.636
Mistakes	16.63 **	<0.001	1.104	22.45 **	<0.001	1.647	5.82	0.125	0.910
Omission	8.27 *	0.001	0.687	11.64 **	<0.001	1.084	3.37	0.429	0.616
Commission	4.91 *	0.002	0.632	5.87 **	0.001	0.761	0.95	1.00	0.901
**Color Trail Test**									
Part A (Standard Score)	1.61	1.00	0.439	33.77 **	<0.001	2.464	35.38 **	<0.001	2.679
Part B (Standard Score)	0.88	1.00	0.142	35.00 **	<0.001	2.572	34.12 **	<0.001	2.458
Digit backwards	2.57 **	<0.001	1.052	6.13 **	<0.001	2.183	3.57 **	<0.001	1.235
Digit sequencing	2.49 **	<0.001	1.084	6.44 **	<0.001	3.104	3.95 **	<0.001	1.084
Letter-number sequencing	4.91 **	0.001	0.256	14.51 **	<0.001	3.101	9.59 **	<0.001	1.591

Note: * *p* < 0.05, ** *p* < 0.001. SD: standard deviation, *t*: *t*-test.

**Table 4:** Lineal multiple regression between capacity to perform basic ADL and attention resources for individuals with ID.

Explanatory Variables	Barthel Index (R^2^ = 0.397)					*p*-Value
	B	95% CI		β	SE	
		Lower Limit	Upper Limit			
Age (years)	−0.357	−0.693	−0.021	−0.279	12.938	0.038
Degree of ID	15.717	7.764	23.670	0.571	12.121	<0.001
“A” test (mistakes)	−0.382	−0.638	−0.126	0.451	11.079	0.004

Note: ID: intellectual disability, R^2^: regression coefficient of determination, B: regression coefficient, CI: confidence interval, β: adjusted coefficient from multiple linear regression analysis, SE: coefficient standard error.

**Table 5:** Lineal multiple regression between capacity to perform IADL and attention resources for individuals with ID.

Explanatory Variables	Lawton and Brody Scale (R^2^ = 0.684)					*p*-Value
	B	95% CI		β	SE	
		Lower Limit	Upper Limit			
“A” test (correct items)	0.057	0.027	0.087	0.295	1.648	<0.001
“A” test (mistakes)	−0.056	−0.082	−0.029	−0.414	1.575	<0.001
Digit forward	−0.343	−0.509	−0.178	−0.469	1.401	<0.001
Digit backwards	0.348	0.099	0.597	0.438	1.321	0.007
Digit sequencing	0.268	0.048	0.488	0.329	1.276	0.018

Note. R^2^: regression coefficient of determination, B: regression coefficient, CI: confidence interval, β: adjusted coefficient from multiple linear regression analysis, SE: coefficient standard error.

## Data Availability

The data can be requested by the scientific community upon reasonable request and within ethical consideration.
